# The global state of GLIM criteria in oncology: a bibliometric analysis of research trends (2019–2025)

**DOI:** 10.3389/fnut.2026.1802280

**Published:** 2026-05-05

**Authors:** Guoqing Zhang, Yishu Lyu, Xinying Liu, Yingyi Chen, Fu Ming, Jiaguo Huang

**Affiliations:** 1 Department of Clinical Nutrition, West China Hospital, Sichuan University, Chengdu, Sichuan, China; 2 Department of Urology, Affiliated Xiaoshan Hospital, Hangzhou Normal University, Hangzhou, Zhejiang, China

**Keywords:** bibliometric analysis, global leadership initiative on malnutrition, inflammation, muscle mass, oncology

## Abstract

**Objectives:**

This study aims to delineate the research landscape and evolutionary trajectory of the GLIM criteria in oncology. By mapping development from initial diagnosis to emerging frontiers, it identifies research hotspots, collaborative patterns, and paradigm shifts to provide a strategic reference for optimizing nutrition management and future oncological inquiries.

**Methods:**

A systematic search was performed in the Web of Science Core Collection and Scopus databases (2019–2025), followed by comprehensive bibliometric analyses and network visualizations using CiteSpace and VOSviewer.

**Results:**

A total of 342 publications were identified, showing a sharp linear increase from 2019 to 94 papers in 2025. China, Spain, Japan, Brazil and Italy led global productivity, with Shi Hanping as the most prolific author, while several European nations achieved higher citation impact. Capital Medical University emerged as the leading institution. The most productive were *Nutrients*, *Clinical Nutrition*, and *Frontiers in Nutrition*. Keyword analysis identified “GLIM criteria” as the central theme, with “overall survival” exhibiting the strongest citation burst. The field has evolved from initial tool validation and body composition analysis toward the increasing application of machine learning-based approaches in prognostic modeling. Current research frontiers emphasize “nutritional therapy” and “precision intervention,” with clinical applications expanding from common gastrointestinal and lung cancers to a broader spectrum of malignancies. This trajectory indicates a maturing research paradigm shifting from basic malnutrition diagnosis toward data-driven, precision clinical intervention.

**Conclusion:**

The GLIM criteria have evolved from a simple diagnostic validation framework toward an emergent role in precision oncology.

## 1 Introduction

Malnutrition is a pervasive issue among cancer patients, impacting not only physiological function and quality of life but also correlating strongly with increased postoperative complications, diminished treatment tolerance, and reduced overall survival (1, 2). It is estimated that approximately 41% of oncology patients suffer from malnutrition, with the prevalence of severe malnutrition reaching as high as 20% (3). However, the burden of malnutrition varies substantially across different oncological populations. The prevalence is particularly high in patients with gastrointestinal, pancreatic, lung, and head and neck cancers, whereas relatively lower rates are observed in breast cancer and hematological malignancies. In addition, advanced disease stage, treatment modalities such as surgery, chemotherapy, radiotherapy, and immunotherapy, as well as patient-related factors including age, comorbidities, and baseline nutritional status further contribute to the heterogeneity of malnutrition risk (4, 5). Cancer-related symptoms such as anorexia and nausea, compounded by metabolic alterations are primary drivers of nutritional deterioration. Consequently, early identification and management of malnutrition are paramount to improving clinical outcomes in this population (6). However, the long-standing global absence of unified, standardized diagnostic criteria has led to heterogeneity in reported prevalence, assessment methodologies, and intervention efficacy across studies, thereby hindering the development of standardized and precision-based clinical nutritional support.

To address this challenge, the Global Leadership Initiative on Malnutrition (GLIM) published consensus-based diagnostic framework in 2019, aiming to establish a universal diagnostic criterion for adult patients (7). The GLIM framework is uniquely suited to identify the nutritional nuances of different oncological populations. The GLIM criteria utilize a two-step approach: first, identifying individuals at high risk via nutritional screening tools, followed by a diagnosis based on three phenotypic criteria (non-volitional weight loss, low body mass index [BMI], and low muscle mass) and two etiologic criteria (reduced food intake/assimilation and inflammation/disease burden). Malnutrition severity can also be graded according to phenotypic criterion (7). Compared to previous assessment tools, GLIM uniquely incorporates muscle mass as a critical phenotypic indicator and integrates etiologic factors, providing a more comprehensive reflection of the pathophysiological mechanisms underlying disease-related malnutrition. This makes it particularly applicable to cancer patients frequently presenting with inflammatory states or metabolic derangements (8, 9). This is especially critical for identifying malnutrition in less conventional presentations within oncology, such as cancer patients with sarcopenic obesity or elderly cancer patients, where traditional BMI-based metrics may fail to accurately detect nutritional risk (10–12).

Since its inception, the validity, predictive value, and implementation feasibility of the GLIM criteria in oncology have garnered extensive academic interest. Numerous studies have confirmed that malnutrition diagnosed via GLIM is significantly associated with increased postoperative complications, infection risk, prolonged hospital stays, and decreased overall survival (13–15). The updated GLIM consensus published in 2025 noted that while further standardization is required regarding muscle mass assessment and inflammatory markers, current evidence supports GLIM as a reliable and pragmatic tool that is increasingly being adopted in clinical practice (16). However, there remains a lack of systematic bibliometric analysis concerning research trends, knowledge structures, collaborative networks, and the academic impact of GLIM within the field of oncology.

Bibliometrics serves as a quantitative method for analyzing the structure, dynamics, and influence of academic literature, revealing research hotspots, evolutionary pathways, and collaborative patterns among core authors and institutions to provide a scientific basis for future research (17). A global bibliometric analysis on the GLIM criteria was recently published in *Frontiers in Nutrition*, which examined overall research trends from 2018 to 2024 using data retrieved from the Scopus database (18). That study provided a comprehensive overview of the global research landscape of GLIM across diverse clinical disciplines. However, the application of GLIM in oncology presents several distinctive challenges that may not be fully captured in a broad global analysis. Cancer patients frequently experience complex metabolic alterations, systemic inflammation, treatment-related nutritional deterioration, and tumor-specific changes in body composition, all of which can significantly influence nutritional assessment and management. Therefore, the present study focuses specifically on oncology-related research involving the GLIM criteria. By restricting the scope to cancer populations, this study enables a more detailed examination of cancer-specific research patterns, including the distribution of tumor types, population subgroups, and clinically relevant outcomes such as muscle mass loss, nutritional intervention strategies, and survival prediction. In addition, this analysis incorporates literature published up to 2025 and integrates data from both the Web of Science Core Collection and Scopus databases, thereby providing an updated and complementary perspective to the previously published global bibliometric study. Although several reviews and meta-analyses on GLIM have been conducted, no study has yet comprehensively mapped the knowledge landscape and developmental trajectory of GLIM in the oncological context from a bibliometric perspective. Therefore, this present study aims to systematically analyze research pertaining to the application of GLIM in cancer patients, with the objective of providing a foundational reference for clinical practice, policy-making, and future research.

## 2 Methods

### 2.1 Literature search strategy

This bibliometric analysis was conducted accordance with the Preliminary guideline for reporting bibliometric reviews of the biomedical literature (BIBLIO) checklist (Supplementary Table S1) (19). A bibliometric analysis was conducted utilizing the Web of Science Core Collection (WoSCC) and Scopus as the primary data sources. To mitigate dynamic biases resulting from database updates, the data retrieval process was completed within a single day. The search spanned from January 1, 2019, to December 31, 2025. Document types were restricted to “articles” and “reviews,” and the language was limited to English. The detailed search strategies are delineated in Supplementary Tables S2, S3.

### 2.2 Literature screening and selection

**Figure 1:**
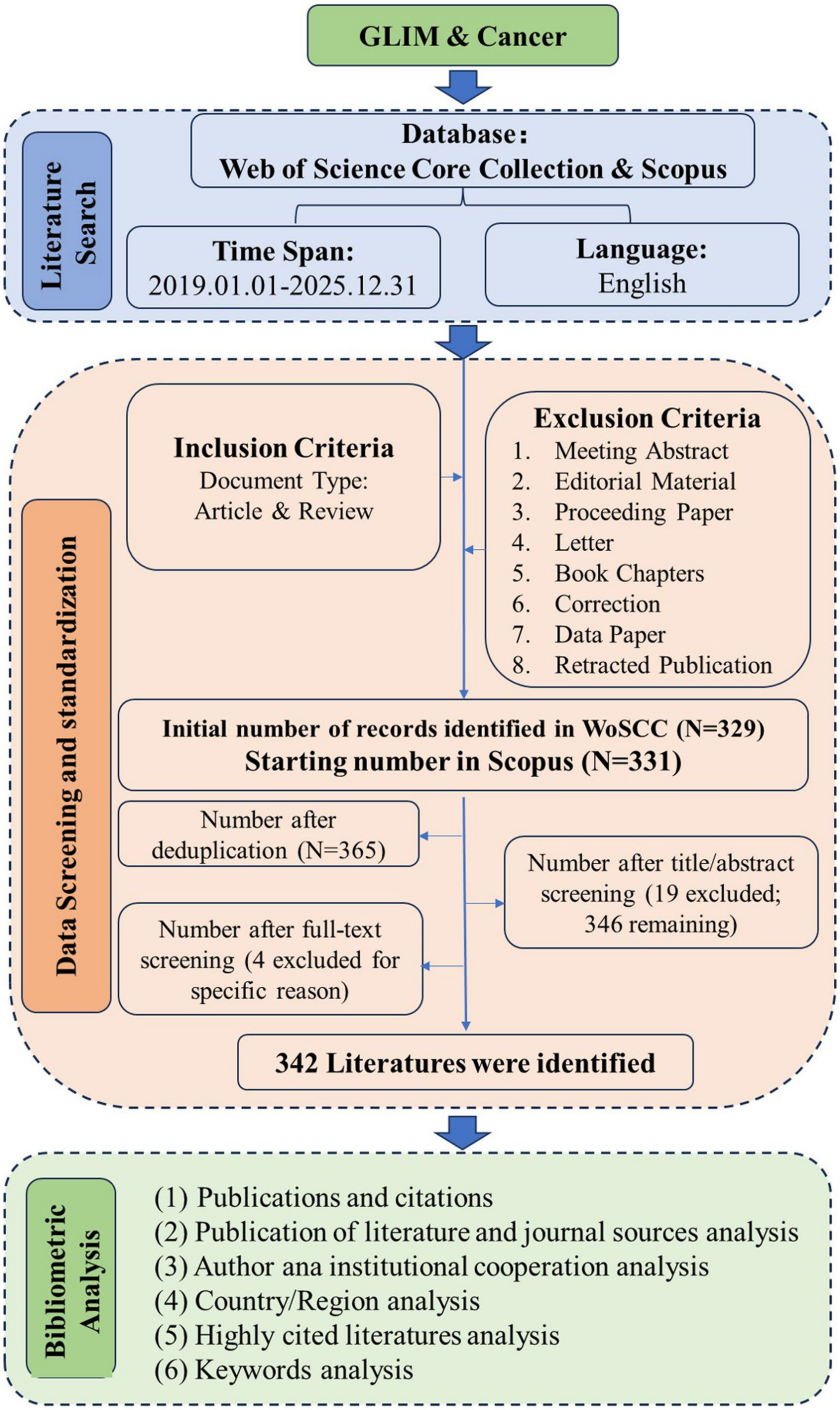
Flow diagram of study selection and data analysis strategies.

The literature screening process is illustrated in Figure 1. Initial records were harvested from WoSCC and Scopus based on the search strategies. To ensure data integrity, all identified records were exported in plain text format for subsequent bibliometric analysis.

Inclusion Criteria: To ensure analytical consistency and reproducibility, studies were included if they met the following criteria: (1) Original research, review articles, or methodological studies; (2) Studies explicitly utilizing GLIM criteria as a diagnostic tool, primary outcome variable, or an analytical framework; (3) Review articles are included only if they contain at least one dedicated section discussing the clinical application or outcomes of GLIM criteria in oncological populations; (4) Study population consists of at least half cancer patients, or includes oncological subpopulations that were stratified and analyzed separately.; (5) Articles published in peer-reviewed journals; (6) Studies published between 2019 and 2025; (7) Availability of full-text versions in English.

Exclusion Criteria: Studies were excluded if they met any of the following: (1) Retracted articles; (2) Duplicate publications (where only the most comprehensive version was retained); (3) Articles referring to GLIM only in passing (e.g., mentioned in introduction or background without methodological application); (4) Commentaries, editorials, letters or opinion pieces lacking original data/analysis; (5) Articles utilizing only predecessor malnutrition frameworks (e.g., SGA, PG-SGA) without GLIM implementation; (6) *in vitro* or animal studies; (7) Gray literature (e.g., dissertations, meeting abstracts without full-text publication); (8) Book chapters; (9) Non-English language publications.

Note on Selection Process: The core inclusion and exclusion criteria were pre-specified to define the study scope and ensure methodological rigor. During the initial screening phase, we refined the criteria for review articles to require at least one dedicated section discussing the clinical application or outcomes of GLIM in oncological populations. A total of 660 records were retrieved from WoSCC and Scopus. After removing duplicates, 365 records remained. Following title and abstract screening, 19 irrelevant articles were excluded. Subsequently, during the full-text screening, four additional studies were excluded for the following reasons: one had no full-text version available; two mentioned the GLIM criteria only in one or two sentences within a single paragraph, failing to provide a dedicated section on their clinical application or outcomes in cancer patients; and one had a study population comprising less than one-third cancer patients, which did not meet our criterion requiring that at least half of the population be cancer patients, or that oncological subpopulations be stratified and analyzed separately.

Screening and Inter-rater Reliability: Two independent researchers (YSL and XYL) performed title, abstract, and full-text screening. To ensure the reliability of the selection process, we calculated the initial inter-rater agreement using Cohen’s Kappa coefficient. Two reviewers independently screened 365 records with an initial agreement of 96.44% (*Kappa* = 0.705), indicating substantial agreement. Discrepancies in 13 records (7 included and 6 excluded) were resolved by a third-party adjudicator (GQZ), resulting in a final inclusion of 342 studies.

### 2.3 Data visualization and statistical analysis

To achieve comprehensive data integration, mining, and visualization, several specialized software was employed, including Microsoft Excel 2019, CiteSpace (v6.4. R1), and VOSviewer (v1.6.20). Microsoft Excel 2019 was utilized for the statistical analysis and visualization of annual publication trends. It was also used to determine the frequency distributions for key contributors and to track the temporal output of the top three journals. For bibliometric visualization, two complementary scientific mapping tools—CiteSpace and VOSviewer—were applied to generate visual representations of authors, institutions, countries/regions, journals, and keywords. These analyses aimed to uncover research collaboration networks, knowledge structures, and developmental trends within the field.

To generate these visual maps, the CiteSpace software was configured with the following parameters: (1) The time span was set from 2019 to 2025 with a 1-year time slice; (2) Node selection employed the g-index, with the scaling factor k set to 50; (3) The Top 50 selection strategy was also applied, extracting the top 50 most cited publications per time slice as nodes; (4) For keyword co-occurrence analysis, cluster labels were extracted via the Log-Likelihood Ratio algorithm; (5) CiteSpace’s built-in Burst Detection algorithm was used to identify keywords with sharp frequency increases over short periods, with the *γ* value set to the 0.1. All other CiteSpace settings and VOSviewer configurations remained at their system defaults. The keyword frequency threshold was set at >10 based on an empirical analysis of keyword distribution, in alignment with established best practices in bibliometric research.

## 3 Results

### 3.1 Global research output: annual trends

**Figure 2:**
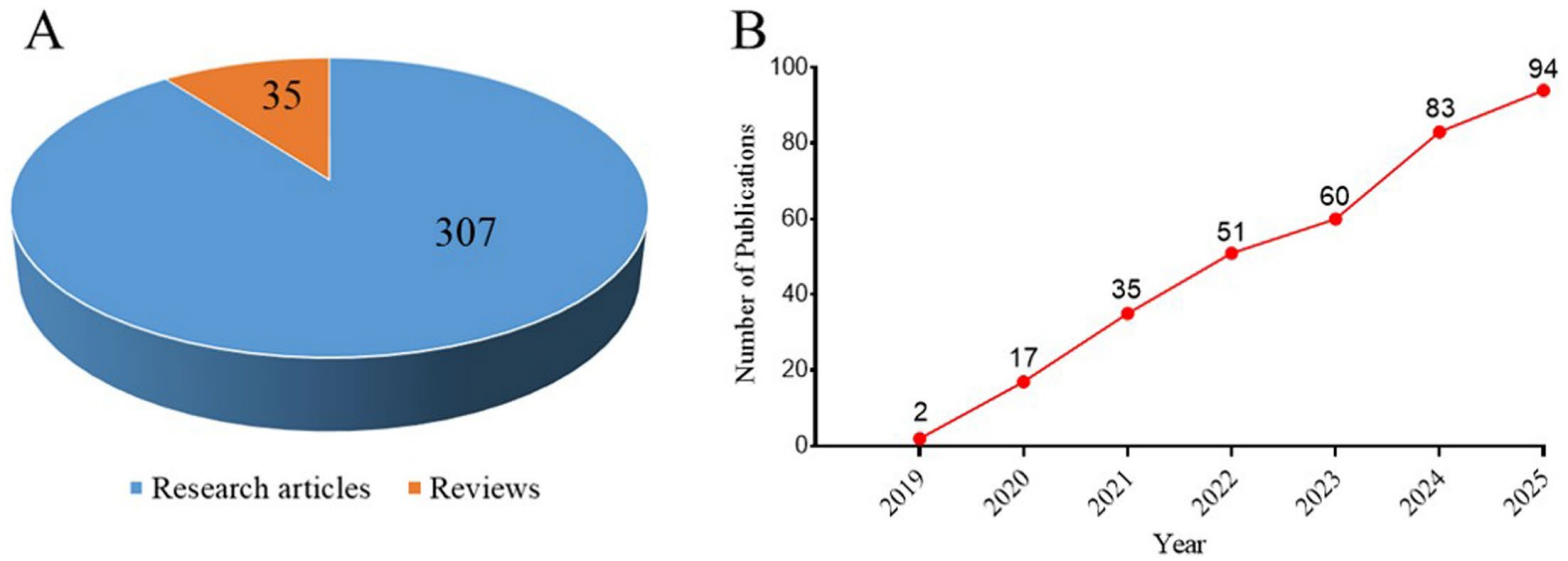
Quantitative analysis of GLIM-related publications in oncology. **(A)** Distribution of publication types. **(B)** Annual growth trajectory of publications from 2019 to 2025.

From 2019 to 2025, a total of 342 publications exploring the association between GLIM criteria and oncology were identified across the WoSCC and Scopus databases. This corpus comprises 307 original research articles and 35 reviews (Figure 2A). This corpus comprises 307 original research articles and 35 reviews. Among the original research articles, study designs included: prospective cohort studies (*N* = 113; 36.8%), retrospective cohort studies (*N* = 82; 26.7%), cross-sectional studies (including retrospective database analyses, *N* = 106; 34.5%), randomized controlled trials (*N* = 5; 1.6%), and other designs (Qualitative research, *N* = 1; 0.3%). Regarding the review articles (*n* = 35), the study designs consisted of 16 narrative reviews (45.7%), 11 systematic reviews with meta-analyses (31.4%) that provided pooled quantitative data, 8 standalone systematic reviews (22.9%) focused on qualitative synthesis. As illustrated in Figure 2B, the annual publication volume surged from a mere 2 papers in 2019 to 94 in 2025, demonstrating a notable sharp growth trajectory. Regression analysis confirmed a robust linear growth trend (*R*^2^ = 0.9942), which significantly outperformed the exponential model (*R*^2^ = 0.8225).

### 3.2 National and institutional publication landscape

A total of 54 countries/regions have contributed to the literature on GLIM and Cancer (Supplementary Table S4). Supplementary Figure S1 illustrates the countries/regions-level distribution of scientific output, stratified by the number of publications. Among these, 13 countries/regions were highly productive, each contributing more than 10 publications. The remaining contributors include 7 countries/regions with 6–9 publications, 19 countries/regions with 2–5 publications, and 15 countries/regions with a single publication. Table 1 lists the leading nations with over 10 publications in this field. China stands at the forefront of global research, with 137 publications over the seven-year period, accounting for 40.1% of the total output. Spain and Japan followed with 42 (12.3%) and 30 (8.8%) publications, respectively. Brazil and Italy each contributed 20 (5.8%) publications, while the output from all other nations remained below this threshold (Table 1 and Supplementary Table S4). China not only produced the highest number of publications but also exhibited the greatest total link strength, underscoring its prominent academic contribution in this area. However, in terms of average citations per article, China (16 citations/article) did not rank highest. Belgium, Australia, Sweden, Italy, and Spain all achieved averages exceeding 20 citations per article, indicating that publications from these countries have attained higher academic impact.

**Table 1:** Top 13 countries/regions for publications.

Rank	Countries/Regions	Documents	Citations	Average citations	Total link strength
1	China	137	2,154	16	1,575
2	Spain	42	826	20	566
3	Japan	30	430	14	375
4	Brazil	20	301	15	381
5	Italy	20	511	26	376
6	USA	19	248	13	280
7	Australia	18	574	32	453
8	Germany	16	273	17	278
9	Sweden	16	485	30	370
10	Scotland	14	271	19	277
11	Netherlands	12	200	17	240
12	Poland	12	99	8	115
13	Belgium	10	400	40	201

In terms of institutional contributions, 684 institutions worldwide have published research related to GLIM in oncology. Supplementary Figure S2 illustrates the institution-level distribution of scientific output, stratified by the number of publications. Among these, 15 institutions were highly productive, each contributing more than 10 publications. The remaining contributors include 14 institutions with 6–9 publications, 136 institutions with 2–5 publications, and 519 institutions with a single publication. Table 2 presents the top 15 institutions ranked by publication count, with Capital Medical University, Peking Union Medical College, and Army Medical University securing the top three positions. Notably, 11 of the top 15 institutions are based in China, with the remaining four located in Spain, Italy, the United Kingdom, and Sweden. These findings further consolidate the observation that Chinese institutions are the most prolific and active participants in this evolving research domain.

**Table 2:** Top 15 institutions for publications.

Rank	Institution	Country	Documents
1	Capital Medical University	China	36
2	Peking Union Medical College	China	29
3	Army Medical University	China	26
4	Zhengzhou University	China	25
5	Jilin University	China	24
6	Wenzhou Medical University	China	23
7	Fujian Medical University	China	18
8	Tongji University	China	13
9	Guangxi Medical University	China	13
10	Universidad de Malaga	Spain	10
11	Sapienza University Rome	Italy	10
12	Wuhan University	China	10
13	University of Glasgow	UK	10
14	Uppsala University	Sweden	10
15	Kunming Medical University	China	10

### 3.3 Analysis of journals

A total of 342 publications were distributed across 119 academic journals. Table 3 highlights the most prolific contributing journals, led by *Nutrients* (*n* = 35), *Clinical Nutrition* (*n* = 34), and *Frontiers in Nutrition* (*n* = 31). Regarding average citations per publication, the *Journal of Parenteral and Enteral Nutrition* emerged as the most frequently cited journal, followed by *Clinical Nutrition* and *Nutrients* (Table 3).

**Table 3:** Top 10 journals for publications.

Rank	Journal	Documents	Citations	Average citations
1	*Nutrients*	35	962	27
2	*Clinical Nutrition*	34	1,121	33
3	*Frontiers in Nutrition*	31	298	10
4	*Clinical Nutrition ESPEN*	23	92	4
5	*Nutrition*	17	405	24
6	*Nutrition in Clinical Practice*	10	47	5
7	*Journal of Parenteral and Enteral Nutrition*	9	307	34
8	*Supportive Care in Cancer*	8	201	25
9	*Nutricion Hospitalaria*	7	68	10
10	*Nutrition and Cancer-an International Journal*	7	98	14

Focusing on the top three journals by publication volume, their annual outputs from 2019 to 2025 revealed distinct phased variations and divergent trends (Supplementary Figure S3). Specifically, the annual output of *Nutrients* followed a fluctuating upward trajectory, peaking in 2024 before a slight contraction. *Clinical Nutrition* experienced a surge in productivity around 2021, which subsequently entered a plateau and showed a downward trend in recent years. In contrast, although *Frontiers in Nutrition* entered this specific field later, it demonstrated robust growth momentum characterized by significant surges in 2022 and 2025.

### 3.4 Author impact analysis

A total of 1,984 researchers has contributed to the body of literature concerning the application of GLIM criteria in oncology. Supplementary Figure S4 illustrates the author-level distribution of scientific output, stratified by the number of publications. Among these, 9 authors were highly productive, each contributing more than 10 publications. The remaining contributors include 23 authors with 6–9 publications, 354 authors with 2–5 publications, and 1,598 authors with a single publication. Table 4 highlights the nine most prolific scholars, each with over 10 publications. Shi, Hanping spearheaded the field with 31 publications, followed by Xu, Hongxia (25 publications), Song, Chunhua (23 publications), and Li, Wei (21 publications). Other prominent contributors include Cui, Jiuwei (13 publications), Yin, Liangyu (11 publications), Guo, Zengqing (11 publications), Zhang, Xi (10 publications), and McMillan, Donald C (10 publications). Notably, the geographic distribution of these authors reveals that the top eight highly productive scholars are all affiliated with Chinese institutions.

**Table 4:** Author impact analysis.

Rank	Author	Documents
1	Shi, Hanping	31
2	Xu, Hongxia	25
3	Song, Chunhua	23
4	Li, Wei	21
5	Cui, Jiuwei	13
6	Yin, Liangyu	11
7	Guo, Zengqing	11
8	Zhang, Xi	10
9	Mcmillan, Donald C	10

### 3.5 Analysis of highly cited references

The top 10 most frequently cited publications are summarized in Table 5. The most cited work is a review by Sanchez-Rodriguez et al., published in 2021, with 215 citations. It is followed by a research article by Shi, Hanping et al. from the same year, published in *Clinical Nutrition*, which received 195 citations. It is noteworthy that while the most-cited document is a review, the remaining nine entries in the top 10 are all original research articles.

**Table 5:** Analysis of highly cited articles/reviews.

Rank	Title	Year	Author	Journal	Citations
1	Sarcopenia, Malnutrition, and Cachexia: Adapting Definitions and Terminology of Nutritional Disorders in Older People with Cancer	2021	Sanchez-Rodriguez, Dolores	*NUTRIENTS*	215
2	The GLIM criteria as an effective tool for nutrition assessment and survival prediction in older adult cancer patients	2021	Shi, Han-Ping	*CLINICAL NUTRITION*	195
3	GLIM Criteria Using Hand Grip Strength Adequately Predict Six-Month Mortality in Cancer Inpatients	2019	Olveira, Gabriel	*NUTRIENTS*	177
4	Malnutrition Screening and Assessment in the Cancer Care Ambulatory Setting: Mortality Predictability and Validity of the Patient-Generated Subjective Global Assessment Short form (PG-SGA SF) and the GLIM Criteria	2020	Barbara S.	*NUTRIENTS*	168
5	Prevalence of malnutrition comparing NRS2002, MUST, and PG-SGA with the GLIM criteria in adults with cancer: A multi-center study	2021	Wan, Hongwei	*NUTRITION*	156
6	Weight loss and BMI criteria in GLIM’s definition of malnutrition is associated with postoperative complications following abdominal resections – Results from a National Quality Registry	2020	Viste, Asgaut	*CLINICAL NUTRITION*	126
7	Impact of malnutrition on early outcomes after cancer surgery: an international, multicenter, prospective cohort study	2023	Marie Carmela	*LANCET GLOBAL HEALTH*	110
8	The effect of malnutrition on mortality in hospitalized patients with hematologic malignancy	2020	Saydam, Guray	*SUPPORTIVE CARE IN CANCER*	109
9	Evaluation of the Global Leadership Initiative on Malnutrition Criteria Using Different Muscle Mass Indices for Diagnosing Malnutrition and Predicting Survival in Lung Cancer Patients	2021	Xu, Hongxia	*JOURNAL OF PARENTERAL AND ENTERAL NUTRITION*	90
10	Agreement between GLIM and PG-SGA for diagnosis of malnutrition depends on the screening tool used in GLIM	2022	Henriksen, Christine	*CLINICAL NUTRITION*	79

### 3.6 Keywords analysis

**Figure 3:**
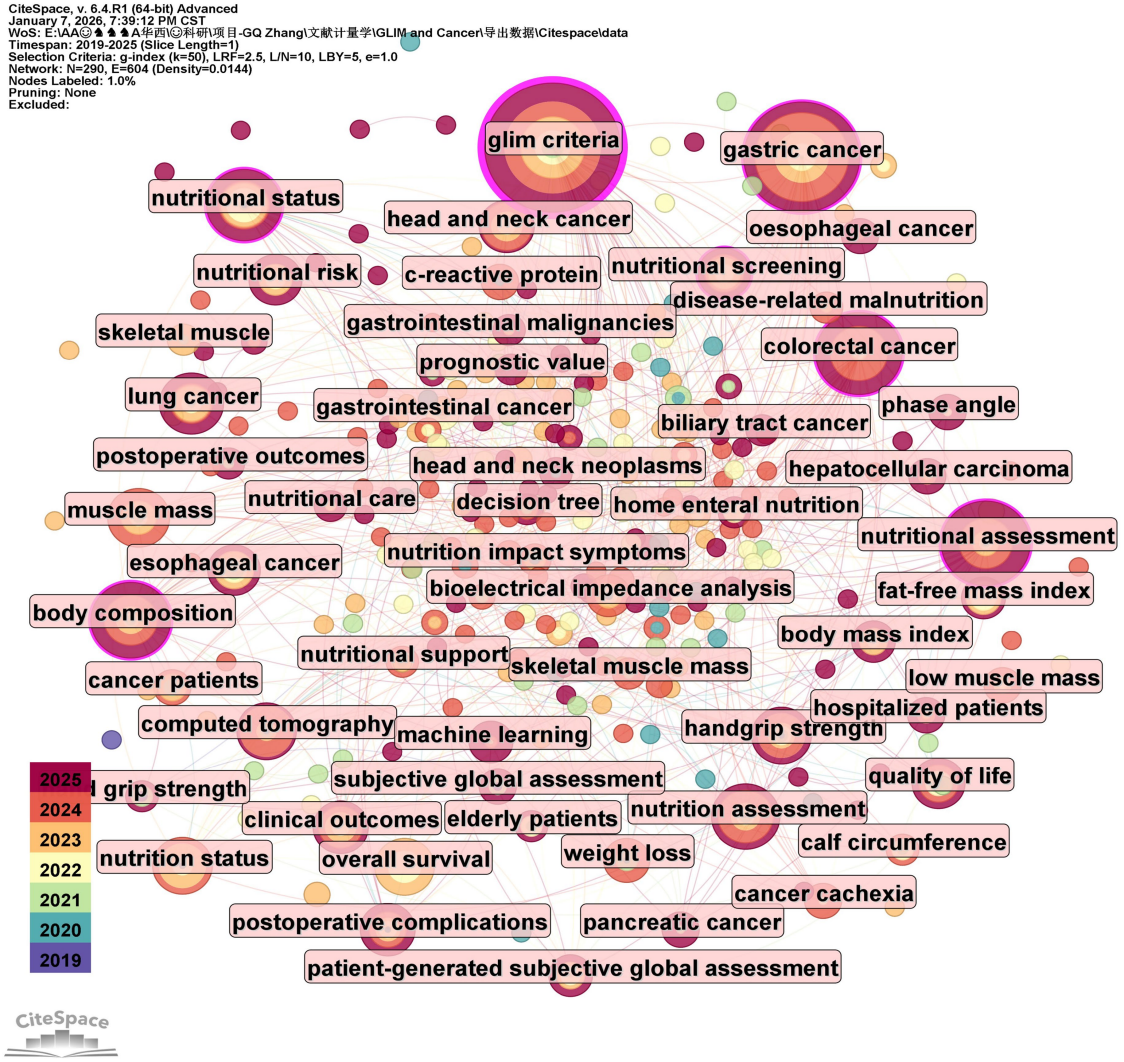
Network visualization map of keywords.

**Figure 4:**
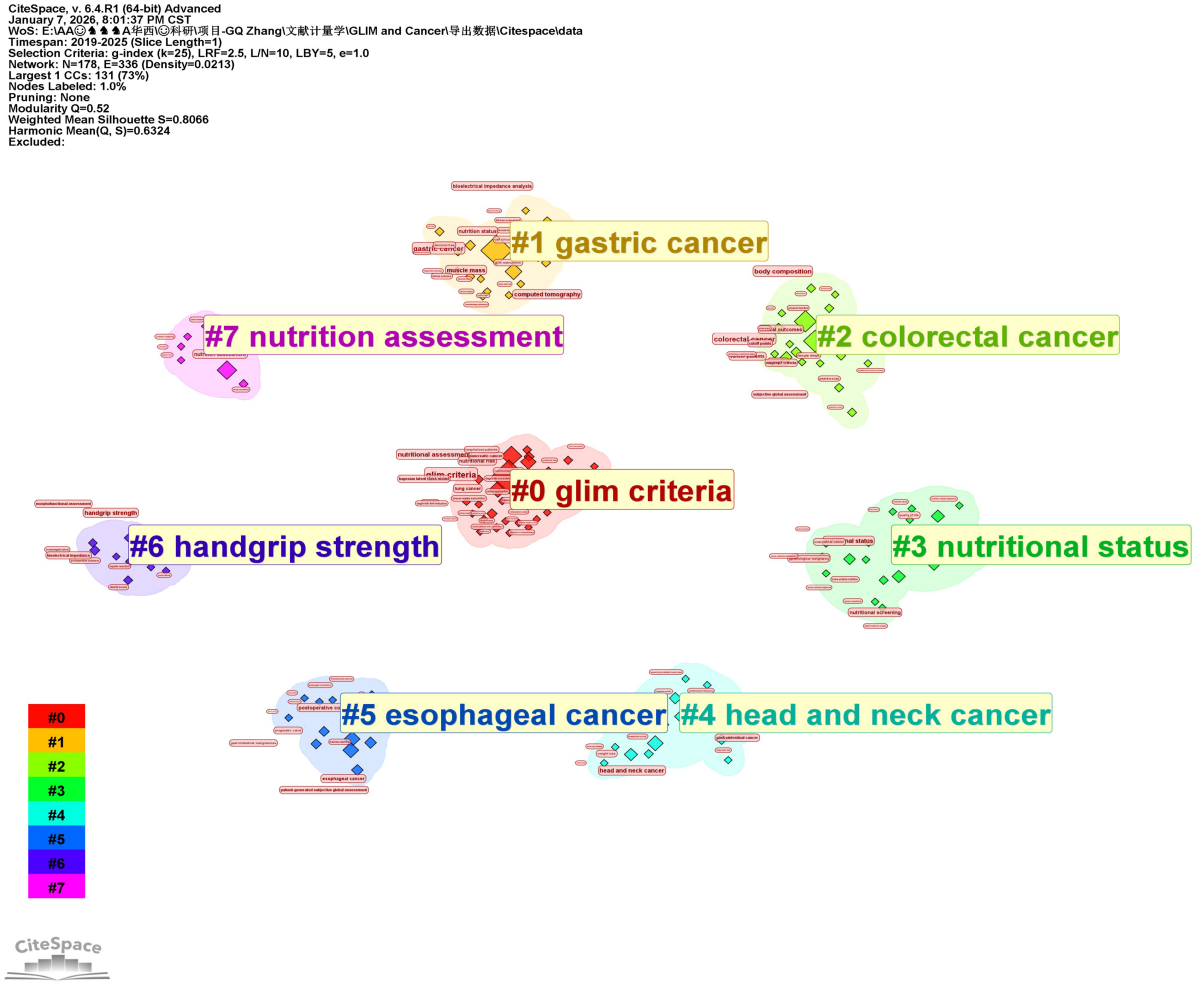
Keyword clusters related to the topic.

Analyzing the core keywords at the intersection of the GLIM criteria and oncology allows for a profound exploration of research hotspots and emerging trends. Based on the 342 included publications, this study used CiteSpace software to extract 290 core keywords. Among these, nine high-frequency keywords appeared more than 10 times (Figure 3): “glim criteria” (49 times), “gastric cancer” (37 times), “colorectal cancer” (22 times), “nutritional assessment” (20 times), “body composition” (18 times), “nutritional status” (16 times), “nutrition assessment” (15 times), “lung cancer” (12 times), and “muscle mass” (12 times). Notably, gastric cancer was the most frequently mentioned tumor type. Cluster analysis of all keywords (Figure 4) revealed eight primary thematic clusters: GLIM criteria, gastric cancer, colorectal cancer, nutritional status, head and neck cancer, esophageal cancer, handgrip strength, and nutrition assessment.

**Figure 5:**
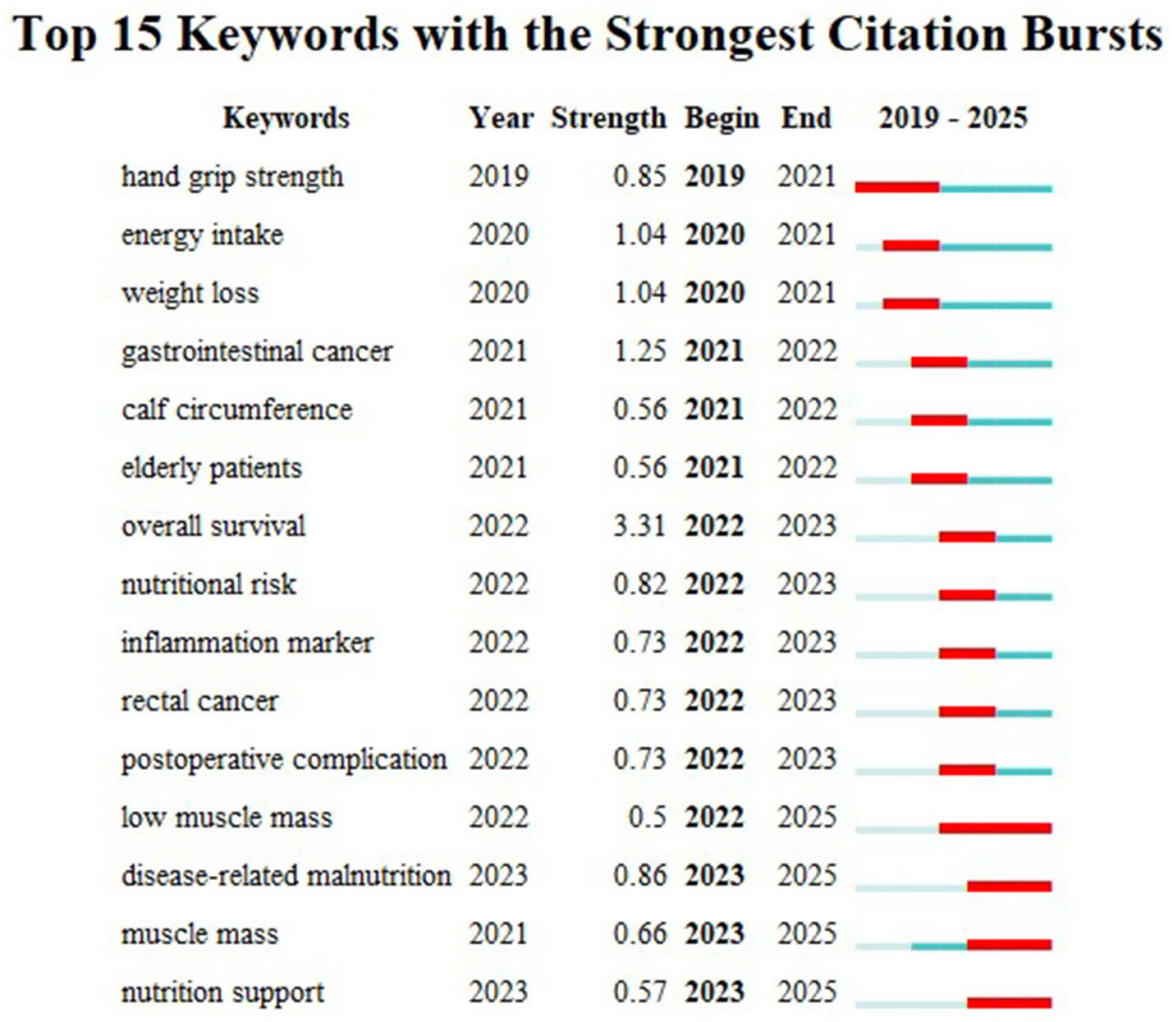
The 15 keywords with the strongest citation bursts.

Figure 5 illustrates the top 15 keywords with the strongest citation bursts. “Overall survival” exhibited the highest burst strength (3.31), underscoring the prognostic significance of GLIM. Furthermore, the burst periods for “low muscle mass,” “disease-related malnutrition,” “muscle mass,” and “nutrition support” have persisted through 2025.

**Figure 6:**
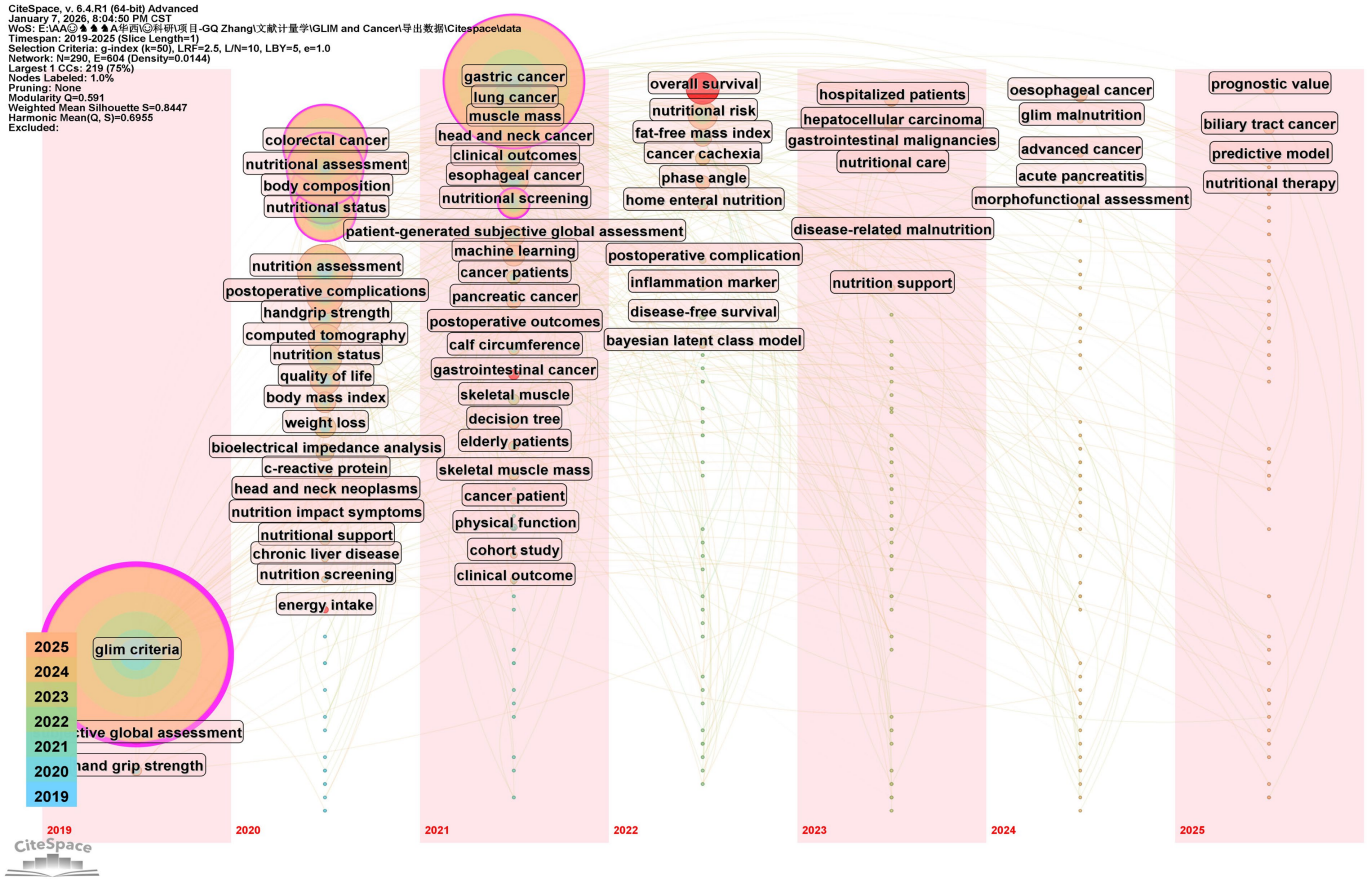
Keyword timezone distribution of GLIM criteria in oncology.

To delineate the progression of research frontiers, a timeline view of core keywords was constructed (Figure 6). The chronological evolution of high-frequency keywords reveals three distinct phases. From 2019 to 2020, keywords primarily included “GLIM criteria,” “malignancies,” “quality of life” and traditional assessment tools such as “PG-SGA,” alongside technical terms like “handgrip strength,” “bioelectrical impedance analysis (BIA),” and “computed tomography (CT).” Between 2021 and 2022, keywords such as “machine learning,” “decision tree,” “elderly populations,” “home enteral nutrition,” “C-reactive protein (CRP)” and “elderly patients” became prominent. In the most recent period (2023–2025), “nutritional therapy,” “nutrition care,” “nutrition support,” “disease-related malnutrition,” “predictive model” and “prognostic value” emerged as high-frequency terms. Furthermore, the longitudinal evolution of keywords indicates that research focus on GLIM has broadened from common malignancies, such as lung and colorectal cancers, to more diverse and complex oncological fields, including hepatocellular carcinoma, gastrointestinal malignancies, and biliary tract cancer.

## 4 Discussion

This study utilizes bibliometric analysis to systematically delineate the research landscape and evolutionary trajectory of the GLIM criteria within the field of oncology. Cancer patients represent a high-risk population for malnutrition due to metabolic dysregulation and/or mechanical obstruction. The GLIM criteria have provided a unified framework for assessing cancer-associated malnutrition and have been widely adopted in the literature. Our analysis of 342 publications from 2019 to 2025 reveals that research in this domain has rapidly transitioned from foundational conceptual explorations to sophisticated, granular investigations, establishing distinct thematic clusters and clear frontier trends.

### 4.1 Global research trends and academic impact

Since its official release in 2019, the GLIM criteria have swiftly emerged as a pivotal focus in oncological nutrition research (13–15, 20). Data from our study demonstrate a substantial increase in related publications within a few years—increasing from a mere 2 papers in 2019 to 94 in 2025. This growth robustly validates the significant value and practical demand for the GLIM framework in unifying diagnostic criteria for malnutrition. This trend not only reflects widespread academic and clinical engagement but also highlights a global imperative for nutritional assessment tools that are standardized, operationally robust, and adaptable across diverse clinical settings.

In terms of scholarly output, China has established a pronounced leading role in this research area, accounting for 40.1% of the total global publications and securing 11 of the top 15 most productive institutions. Scholars represented by Professor Shi, Hanping rank among the world’s leaders in research productivity. This achievement is largely attributed to China’s proactive promotion of standardized nutritional diagnosis and treatment in oncology. A cornerstone of this progress is the “Investigation on Nutrition Status and Clinical Outcome of Common Cancers (INSCOC)” project—a large-scale, multicenter initiative led by Professor Shi, Hanping. Covering 18 common malignancies across hundreds of hospitals and encompassing tens of thousands of patients, this database has accumulated a wealth of clinical nutrition data. Such a robust data infrastructure has provided critical support for the rapid implementation of GLIM assessments, significantly catalyzing the validation and application of these criteria in China’s clinical landscape (21–24).

While China maintains a clear quantitative lead in total publications, a discernible disparity exists in citational impact compared to countries such as Belgium, Australia, and Sweden. This structural contrast suggests a divergence in research strategies: while Chinese research has demonstrated remarkable leadership in the large-scale clinical validation of GLIM criteria across diverse malignancies, European nations have historically focused on foundational methodological innovation and high-impact theoretical frameworks (25, 26). For instance, influential reviews and methodological treatises from European cohorts during the early adoption phases have achieved higher average citations, reflecting a strategy centered on shaping the field’s conceptual evolution. Bridging this gap by transitioning from rapid clinical application to more original, mechanism-oriented research will be pivotal for further enhancing the global influence of China’s extensive research output.

Notably, Brazil also emerged as a significant contributor, ranking fourth globally with 20 publications. This level of productivity highlights the active development of clinical nutrition research in Brazil and the growing adoption of GLIM criteria in oncological practice. However, the current estimate (5.8%) may still underestimate Brazi’s overall research contribution. This potential underrepresentation may be related to two factors. First, some regional or national journals from Latin America may have limited coverage in major international databases. Second, a proportion of clinically relevant studies may be published in Portuguese-language journals, which are often excluded from English-restricted bibliometric analyses. Taken together, these factors suggest that the actual dissemination and clinical implementation of GLIM criteria within Portuguese-speaking research communities may be broader than indicated by the present dataset.

Taken together, these findings point to a complementary yet imbalanced research landscape, characterized by high-volume evidence generation in certain regions and higher-impact methodological contributions in others. Strengthening international collaboration, particularly between high-output and high-impact research clusters, may help bridge this divide by integrating large-scale clinical validation with methodological innovation. Such efforts could enhance the translational value of GLIM criteria in oncology and accelerate their incorporation into clinical practice guidelines. Furthermore, future studies incorporating co-authorship network analyses may provide deeper insights into global collaboration patterns and the extent of research integration in this field.

Regarding the distribution of journals, research findings are predominantly concentrated in specialized nutrition journals such as *Nutrients*, *Clinical Nutrition*, and *Frontiers in Nutrition*, as well as authoritative journals in parenteral and enteral nutrition like the *Journal of Parenteral and Enteral Nutrition*. Additionally, a portion of the literature has been integrated into oncology-specific journals. This distribution pattern clearly indicates that the application of GLIM criteria has achieved a distinct disciplinary focus, with findings progressively modernizing both the conceptual frameworks and practical applications of nutritional assessment and supportive care in oncology.

An intriguing pattern emerging from this analysis is the pronounced geographic and institutional concentration of GLIM-oncology research. A substantial proportion of publications originate from a relatively limited number of countries, institutions, and highly productive authors. While this concentration facilitates rapid accumulation of clinical evidence and large-scale validation across diverse cancer types, it may also increase the likelihood of methodological overlap or redundancy, particularly when studies focus on similar validation frameworks in different tumor populations.

To clarify the distinctions between our study and the recent global bibliometric analysis by Xu et al. (18), a comparative summary is provided in Supplementary Table S5. As shown in the table, the previous study analyzed 729 publications retrieved from the Scopus database between 2018 and 2024 and provided a broad overview of global GLIM-related research across multiple clinical fields. In contrast, the present study focused specifically on oncology-related research and incorporated literature published up to 2025 from both the WoSCC and Scopus databases. Despite differences in scope and methodology, several consistent patterns were observed between the two analyses. For example, both studies identified China, Spain, Japan, Brazil among the leading contributor to GLIM-related research and highlighted journals such as *Nutrients, Clinical Nutrition* and *Frontiers in Nutrition* as major publication venues. These similarities confirm the overall robustness of the global research trends previously reported. However, restricting the analysis to oncology literature revealed several important domain-specific characteristics that are less apparent in global analyses. First, oncology-focused research demonstrates a pronounced emphasis on gastrointestinal malignancies, particularly gastric and colorectal cancers, which are frequently associated with severe malnutrition. Second, keywords related to body composition, such as muscle mass and sarcopenia, appear more prominently in oncology studies, reflecting the clinical importance of cancer-associated muscle wasting. Third, prognostic indicators, especially overall survival, emerge as major research hotspots, indicating that GLIM criteria are increasingly used not only for nutritional diagnosis but also for predicting clinical outcomes in cancer patients. Overall, these findings suggest that an oncology-focused bibliometric approach provides additional insights beyond those obtained from a global analysis, enabling a more detailed understanding of tumor-specific research patterns, prognostic applications, and emerging nutritional intervention strategies in cancer populations.

### 4.2 Evolution of research hotspots

Early research on the GLIM criteria primarily focused on gastric, colorectal, esophageal, and head and neck cancers (27, 28). These studies highlighted the severe nutritional challenges faced by patients with gastrointestinal malignancies due to mechanical obstruction and metabolic alterations, reflecting the significant global burden of these diseases. Given their high risk of malnutrition, treatment-related metabolic disturbances, and poor prognostic profiles, these patients’ cohorts have served as vital clinical populations for the validation and application of the GLIM framework.

A central focus in this field remains the comparative analysis of the diagnostic validity and prognostic value of GLIM versus traditional tools, such as the PG-SGA. This includes comparisons of diagnostic agreement, sensitivity, specificity, complication rates, and mortality (29–31). The PG-SGA, designed specifically for cancer patients, offers the advantage of comprehensiveness by combining patient self-assessment—covering weight change, dietary intake, symptoms, and functional capacity—with professional evaluation, including physical examination and metabolic requirements (32). In contrast, the GLIM criteria, which utilize muscle mass as a core phenotypic indicator and integrate etiological factors, are characterized in the literature as a more objective, mechanism-based approach, demonstrating potential for broad cross-population comparability. Although preliminary evidence supports the applicability of GLIM in various cancers, diagnostic heterogeneity persists across different tumor types (33–36). Consequently, large-scale, multicenter prospective trials have been suggested as a next step to further refine its diagnostic performance and prognostic precision across diverse oncological populations.

Keywords such as “body composition,” “muscle mass,” “handgrip strength,” and “inflammation marker” correspond directly to the phenotypic and etiological components of the GLIM criteria. In the hypermetabolic state induced by cancer, skeletal muscle depletion is a hallmark of cachexia. Early research established a methodological foundation by quantifying “reduced muscle mass”—a core GLIM phenotype—using handgrip strength, CT-derived skeletal muscle index, and BIA-derived skeletal muscle index. Research indicates that the selection of phenotypic criteria varies among patients with different body compositions. In obese cancer patients, reliance solely on low BMI as a phenotypic criterion may fail to effectively identify malnutrition, making measurements of weight loss percentage and muscle mass particularly critical (37). Furthermore, employing alternative methods or modified measurement tools for specific cancer types may offer greater feasibility and clinical value. In gastric cancer, left calf circumference has been identified as a feasibility as a proxy for muscle mass (38), while incorporating functional metrics like handgrip strength into a modified GLIM framework is reported to enhance the prediction of postoperative outcomes in hepatocellular carcinoma (39).

Within the GLIM diagnostic framework, the determination of etiological criteria is a key thematic area for elucidating the pathophysiological mechanisms underlying cancer-related malnutrition. Although cancer is generally considered a disease associated with chronic inflammation, the inflammatory burden varies by cancer type and stage. Therefore, the literature suggests that a more precise definition of the inflammation criterion may potentially enhance the sensitivity of malnutrition identification (40). By incorporating objective inflammatory markers—such as the inflammatory burden index, CRP level, neutrophil-to-lymphocyte ratio (NLR), and albumin—the GLIM criteria can more sensitively reflect metabolic disturbances in the context of cancer. Indeed, studies have reported that GLIM criteria integrated with specific inflammatory biomarkers exhibit enhanced predictive power for both short- and long-term survival compared to the original version (41). Future research should focus on developing cancer-specific phenotypic thresholds and etiological modifications to construct precision nutrition models closely aligned with clinical outcomes.

As the field has evolved, the scope of research has expanded from common malignancies (e.g., lung, gastric, colorectal) to a wider range of malignancies, including pancreatic cancer, biliary tract cancer, hepatocellular carcinoma, and hematological neoplasms (39, 42–44). Simultaneously, significant attention has shifted toward the vulnerable geriatric oncology population (25, 45). With the global aging trend, elderly patients represent an increasing proportion of cancer cases. Their diminished physiological reserve, multiple comorbidities, and altered metabolic and immune profiles often lead to reduced tolerance for oncological therapies, exacerbating malnutrition, functional decline, and impaired quality of life (25, 46). Since quality of life is a critical metric for medical outcomes and influences treatment adherence and decision-making, exploring the nexus between nutritional status and quality of life in older adults is a prominent focus for developing tailored, patient-centered care models that integrate geriatric medicine with oncology.

In recent years, the research focus in this domain has shifted gradually from validation of diagnostic criteria toward nutritional intervention and precision prognosis assessment. The emergence of keywords such as “nutritional support” and “nutritional therapy” signifies an evolution toward therapeutic intervention. Notably, the rise of terms like “machine learning,” “decision tree,” “predictive model,” and “Bayesian latent class model” reflects an evolving research interest in data-driven, intelligent forecasting. The construction of multidimensional models integrating GLIM diagnoses with clinical data suggests a growing academic interest in moving toward personalized and precision-based nutritional assessment (21, 23, 47, 48). Finally, “Overall Survival” stands out as the keyword with the highest burst strength, underscoring a global academic consensus that malnutrition defined by GLIM is frequently identified as an independent risk factor for postoperative complications and decreased survival (13, 14, 49). Looking forward, the development of intelligent prognostic models, combined with individualized nutritional intervention strategies spanning the entire care continuum, is currently emerging as a prominent research direction with the potential to inform clinical decision-making.

### 4.3 Research translation and clinical implementation

While the present study demonstrates a rapid expansion of research on GLIM in oncology, particularly in areas such as machine learning and prognostic modeling, the translation of these findings into routine clinical practice remains incompletely characterized. Several factors may contribute to a potential gap between research activity and clinical implementation. First, although the GLIM criteria were developed to standardize the diagnosis of malnutrition, their adoption in oncology settings is not yet universal, and considerable heterogeneity persists across institutions and regions. Second, many of the emerging research directions identified in this study, such as machine learning–based predictive models, often rely on advanced data infrastructure and computational resources, which may limit their applicability in resource-constrained clinical environments. Third, despite the growing body of literature, major international oncology guidelines have not yet uniformly incorporated GLIM as a mandatory standard for nutritional assessment, suggesting that further high-quality evidence and consensus-building efforts are required.

Collectively, these findings highlight potential research–practice gap in the field of oncological nutrition. Future studies should prioritize translational research focusing on real-world implementation, including the evaluation of barriers to GLIM adoption, the integration of predictive models into clinical workflows, and the development of accessible tools to facilitate bedside application. Additionally, efforts to align research outputs with clinical guidelines and to ensure equitable implementation across diverse healthcare settings will be essential to maximize the clinical impact of GLIM-based approaches.

## 5 Limitations

Several limitations of this study should be acknowledged. First, regarding language scope, the analysis was restricted to English-language publications. Although English has become the dominant language of international scientific communication, this criterion may have introduced language bias. Clinical nutrition research has active communities in several non-English-speaking regions, particularly in Ibero-American countries such as Spain, Brazil, and other Latin American nations, where studies may also be published in Spanish or Portuguese. Consequently, the exclusion of non-English literature could potentially result in a relative underestimation of research output from these regions and may slightly influence the geographical distribution of publications. Furthermore, although the WoSCC and Scopus databases provide broad international coverage, both databases are known to exhibit a certain preference for English-language journals, which may further contribute to the underrepresentation of regional publications. Given that the GLIM criteria were developed through international collaboration and are applied worldwide, relevant research may exist in multiple languages. Nevertheless, it is worth noting that most high-impact journals indexed in WoSCC and Scopus publish primarily in English, and English-language publications generally represent the core of globally disseminated scientific research. Therefore, while the language restriction may introduce some bias, its overall influence on the identification of major research trends is likely to be limited. Future bibliometric studies could incorporate multilingual databases and conduct sensitivity analyses across different languages to provide a more comprehensive evaluation of global research output. Additionally, a temporal limitation concerns the data completeness for the year 2025. Since database indexing often lags behind the actual publication date, the bibliometric data for 2025 collected in early 2026 may not fully capture all publications from the final quarter of the year. While this study provides a reliable snapshot of the trajectory up to this point, the absolute citation counts and the total number of publications for 2025 should be interpreted as a conservative estimate. However, given that bibliometric analysis focuses on identifying long-term structural trends rather than real-time tracking, this partial indexing is unlikely to significantly alter the identified research clusters or the overall evolutionary direction of GLIM-oncology research. Another key limitation is the absence of a formal methodological quality assessment of the 342 included publications. Bibliometric analysis, by design, focuses on mapping research trends, citation patterns, and the knowledge landscape rather than synthesizing evidence robustness. Consequently, publications with significant variations in design, sample size, or statistical rigor are treated equivalently in our analysis. Furthermore, high citation frequency does not necessarily equate to scientific validity, as influential papers may attract citations from both supporting and refuting perspectives. Therefore, this study reflects the quantity and developmental trajectory of research production rather than an evaluation of evidence strength. Readers seeking definitive clinical guidance on the validity of GLIM criteria should consult specific systematic reviews and meta-analyses of diagnostic accuracy, rather than relying solely on these bibliometric patterns. From a procedural standpoint, a limitation of this analysis is the absence of a previously registered protocol, which reduces methodological transparency and increases the potential risk of bias in the selection of results. Additionally, regarding the analytical tools employed, this analysis utilized CiteSpace and VOSviewer; however, complementary analysis with the bibliometrix R package was not conducted. Future studies might triangulate findings across all three mapping tools to ensure a more robust visualization of the field.

## 6 Conclusion

This study systematically delineates the research evolution of the GLIM criteria in the field of oncology. Since their inception in 2019, the GLIM criteria have provided a standardized framework for the standardized diagnosis of cancer-associated malnutrition by integrating phenotypic and etiological indicators, sparking a rapid expansion in scholarly inquiry. Initial research focused predominantly on validating the diagnostic efficacy of GLIM in high-risk populations—specifically those with gastric and colorectal cancers—and comparing its performance against established tools like the PG-SGA. Subsequently, the research focus shifted toward the refinement of core metrics, including skeletal muscle quantification methods and the identification of sensitive inflammatory biomarkers. Currently, the literature reflects a growing interest in shifting toward clinical intervention and precision prognostication, characterized by the emergence of machine learning and multidimensional predictive models. While bibliometric trends highlight the independent predictive value of GLIM for patient outcomes in various studies, this analysis primarily maps research production patterns and identifies emerging themes (machine learning, precision prognosis, specific malignancies) rather than offering validated clinical recommendations. Moving forward, potential research directions identified in the literature include multicenter clinical validation, the enhancement of muscle quality assessment feasibility, and the integration of GLIM into intelligent, comprehensive nutrition management pathways. Clinical practitioners seeking evidence-based guidance should prioritize consultation of systematic reviews and meta-analyses over publication frequency trends.

## Data Availability

The raw data supporting the conclusions of this article will be made available by the authors, without undue reservation.
