# Supplementary material for: The global state of GLIM criteria in oncology: a bibliometric analysis of research trends (2019–2025)
Source: Front Nutr. 2026 May 5;13:1802280. doi: 10.3389/fnut.2026.1802280 (PMC13183560; doi:10.3389/fnut.2026.1802280)
Supplement: Supplementary file 1 [file Table_1.docx]

**Supplementary materials**

**The Global State of GLIM Criteria in Oncology: A Bibliometric Analysis of Research Trends (2019-2025)**

Guoqing Zhang^1^, Yishu Lyu^1^, Xinying Liu^1^, Yingyi Chen^1^, Fu Ming^1^, Jiaguo Huang^2*^

^1^ Department of Clinical Nutrition, West China Hospital, Sichuan University, Chengdu, Sichuan, China.

^2^ Department of Urology, Affiliated Xiaoshan Hospital, Hangzhou Normal University, Hangzhou, Zhejiang, China.

* Correspondence:

[13735526508@163.com](mailto:13735526508@163.com) (Jiaguo Huang)

**Table 1** The BIBLIO checklist for reporting the bibliometric reviews of the biomedical literature

| Section/Topic | Item No. | Checklist item | Reported in section |
| --- | --- | --- | --- |
| Title |  |  |  |
| Identification | 1 | Identify the report as a bibliometric review in the title. | Title |
| Issues/topics | 2 | Indicate the key issues/topics under investigation and coverage of time period. | Title |
| Abstract |  |  |  |
| Structured summary | 3 | Structured summary including (as applicable): background, methods, results (key findings) and conclusions. | Abstract |
| Introduction/ Background |  |  |  |
| Justification/ Rationale/ Explanation | 4 | Present review of existing knowledge and epidemiological information. | Introduction |
| Objectives | 5 | Statement of the objective (s) or question (s). | Introduction |
| Methods |  |  |  |
| Search engines (data sources) | 6 | Describe all information sources (such as electronic databases, contact with study authors, trial registers or other grey literature sources). | Methods |
| Search strategy | 7 | Keywords and systematization criteria (date of search, language, type of document) for the search. | Methods |
| Time period | 8 | The period that the review covers and the justification. | Methods |
| Eligibility criteria | 9 | Describe all inclusion and exclusion criteria; languages; study design, type of publication and time period. | Methods |
| Data refinement (data selection procedure) | 10 | Remove the irrelevant articles; inspection to eliminate duplicate and unrelated articles (after evaluation of the title, abstract and content). | Methods |
| Quality assessment (optional) | 11 | Assessment of papers by three authors and the use of assessing checklists. | Methods |
| Data synthesis | 12 | Describe the methods used for summarizing, handling, synthesis, tabulations or schematic displays. Describe how the data were analysed. | Methods |
| Results |  |  |  |
| Descriptive findings (statistics) | 13 | - Provide details of the search and selection process in a flow diagram.  - Number of citations retrieved (number of publication, year of publication, type of documents, country of publication, articles with the highest impact, most impactful authors, most impactful articles, authors with the highest production, top journals, top institutions, …) | Results Figure 1 |
| Schematic map and trend | 14 | Summarize and/or present the schematic maps and trends using an appropriate software to present citations, journals, authors, top journals, time trends, emerging literature, and any relevant indicators (as applicable) [1-5]. | Results Figures 2-6; Tables 1-5 |
| Tabulation and summarizing the findings | 15 | General recommendation: Studies under consideration could be summarized and organized by different subtitles and different scenarios. Regardless, results need to be presented in separate tables covering each subtitle. The followings are some options that could help to summarize the findings.  *Option 1:*  - Start the presentation with a historical view [when and who first published on the topic].  - Report on review papers. The result should be listed in a separate table. Also, specify the review type (scoping review, narrative review, systematic review, and meta-analysis).  - Summarize the findings according to the study designs and main study types.  *Option 2:*  - Start the presentation with a historical view [when and who first published on the topic].  - Report on review papers. The result should be listed in a separate table. Also, indicate the review type (scoping review, narrative review, systematic review, and meta-analysis) should be specified.  - Summarize the findings according to outcome measures or populations. For example, see [6].  *Option 3:*  - Start the presentation with a historical view [when and who first published on the topic].  - Report on review papers. The result should be listed in a separate table. Also, specify the review type (scoping review, narrative review, systematic review, and meta-analysis).  - Summarize the findings according to concept [7].  *Option 4.*  - Start the presentation with a historical view [when and who first published on the topic].  - Report on review papers. The result should be listed in a separate table, and also specify the review type (scoping review, narrative review, systematic review, and meta-analysis).  - Summarize the findings according to different subtitles relevant to the main topic [8]. | Results Figures 2-6; Tables 1-5 |
| Synthesis of findings | 16 | Synthesize the findings as much as possible, find the gap, and propose a model, hypothesis, etc. (if applicable). | Results Figures 2-6; Tables 1-5 |
| Discussion |  |  |  |
| Summary of evidence | 17 | Summarize the main findings. The findings should be presented in more "general" or "accessible" terms. | Discussion |
| Interpretation | 18 | Include interpretation consistent with results. Explanations for observed outcomes, similarities, and differences reported would be essential. | Discussion |
| Strengths and limitations | 19 | Discuss the strengths and limitations. | Limitations |
| Conclusion(s) | 20 | Provide a general interpretation of the results with respect to the review questions and objectives, as well as potential implications. | Conclusion(s) |
| 1. McDougal L, Dehingia N, Cheung WW, Dixit A, Raj A. COVID-19 burden, author affiliation and women's well-being: A bibliometric analysis of COVID-19 related publications including focus on low-and middle-income countries. eClinicalMedicine 2022; 52: 101606.  2. Henstock L, Wong R, Tsuchiya A, Spencer A. Behavioral theories that have influenced the way health state preferences are elicited and interpreted: A bibliometric mapping analysis of the ttime trade-off method with VOSviewer visualization. Front Health Serv 2022; 2: 848087.  3. Bodea F, Bungau SG, Negru AP, Radu A, Tarce AG, Tit DM, et al. Exploring new therapeutic avenues for ophthalmic disorders: Glaucoma-related molecular docking evaluation and bibliometric analysis for improved management of ocular diseases. Bioengineering 2023; 10(8): 983.  4. Sang XZ, Wang CQ, Chen W, Rong H, Hou LJ. An exhaustive analysis of post-traumatic brain injury dementia using bibliometric methodologies. Front Neurol 2023; 14: 1165059.  5. Ramli MI, Hamzaid NA, Engkasan JP, Usman J. Respiratory muscle training: a bibliometric analysis of 60 years’ multidisciplinary journey. Biomed Eng Online 2023; 22(1): 50.  6. Akosman I, Kumar N, Mortenson R, Lans A, De La Garza Ramos R, Eleswarapu A,et al. Racial differences in perioperative complications, readmissions, and mortalities after elective spine surgery in the United States: A systematic review using AI-assisted bibliometric analysis. Glob Spine J 2023: 21925682231186759.  7. Tavousi M, Mohammadi S, Sadighi J, Zarei F, Kermani RM, Rostami R, Montazeri A. Measuring health literacy: A systematic review and bibliometric analysis of instruments from 1993 to 2021. Plos One 2022; 17(7): e0271524.  8. Montazeri A. Health-related quality of life in breast cancer patients: A bibliographic review of the literature from 1974 to 2007. J Exp Clin Cancer Res 2008; 27: 32. | | | |

**Table 2.** Search Strategies in the Web of Science Core Collection

| Step | Formular |
| --- | --- |
| #1 | TS=(GLIM) OR TS= (Global Leadership Initiative on Malnutrition) |
| #2 | TS= (cancer* OR neoplas* OR oncolog* OR metasta* OR malignan* OR adenoma* OR tumor* OR tumour* OR adenocarcinoma* OR blastoma* OR carcinogen* OR carcinom* OR carcinosarcoma* OR chordoma*) |
| #3 | #1 AND #2 |

**Table 3.** Search Strategies in the Scopus

| Formular |
| --- |
| (TITLE-ABS-KEY (glim) OR TITLE-ABS-KEY (global AND leadership AND initiative AND on AND malnutrition)) AND TITLE-ABS-KEY (cancer* OR neoplas* OR oncolog* OR metasta* OR malignan* OR adenoma* OR tumor* OR tumour* OR adenocarcinoma* OR blastoma* OR carcinogen* OR carcinom* OR carcinosarcoma* OR chordoma*) |

**Table 5.** Comparison between the previously published global bibliometric analysis of GLIM research and the present oncology-focused study

| Aspect | Current study | Xu et al. study (13) |
| --- | --- | --- |
| Research Scope | GLIM research specifically in oncology | Global GLIM research across multiple clinical fields |
| Times Period | 2019-2025 | 2018-2024 |
| Data Sources | WoSCC+Scopus | Scopus |
| Number of publications | 342 (Oncology-specific) | 729 |
| Analytical tools | VOSviewer and CiteSpace | VOSviewer, CiteSpace, and the online platform Bioinformatics |
| Keyword analysis | Cancer-specific themes | General GLIM research themes |
| Clinical focus | Cancer-related malnutrition, prognosis, and nutritional interventions | Broad malnutrition research |
| Additional analyses | Tumor-type distribution, prognostic indicators, and oncology-specific research evolution | Global research landscape |


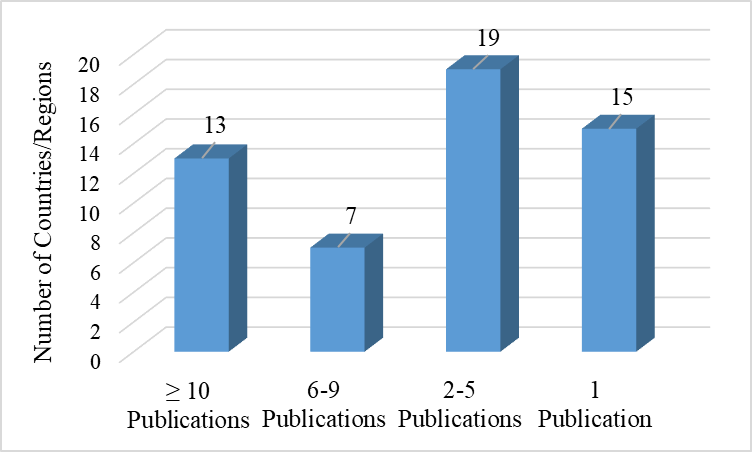


Figure 1 Frequency distribution of countries/regions by publication volume


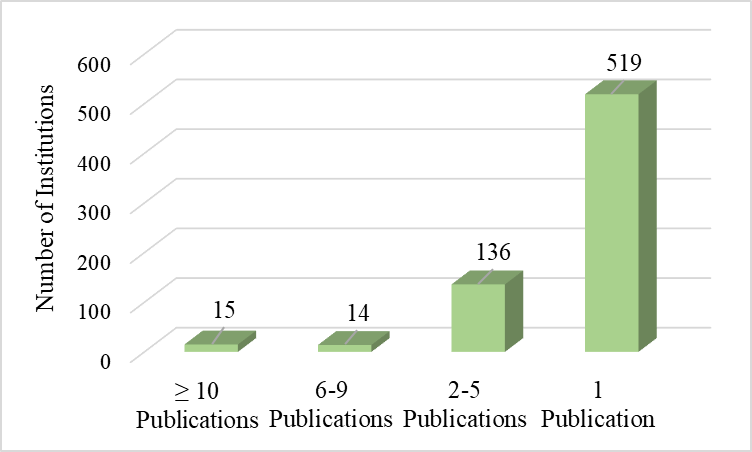


Figure 2 Frequency distribution of institutions by publication volume


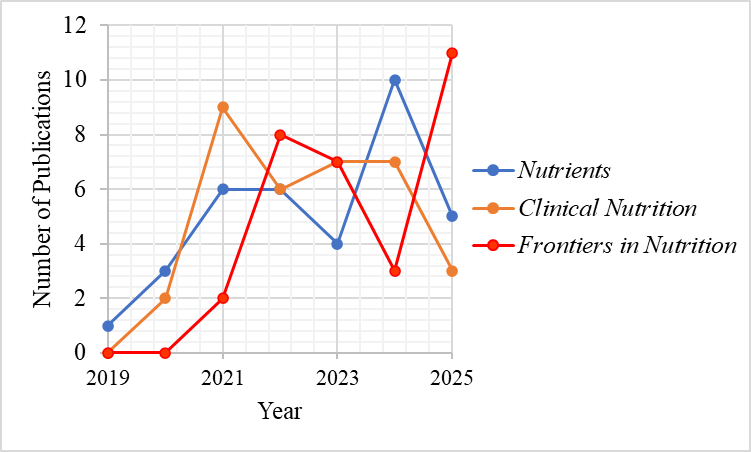


Figure 3 Annual publication trends of the top 3 journals by article volume (2019–2025)


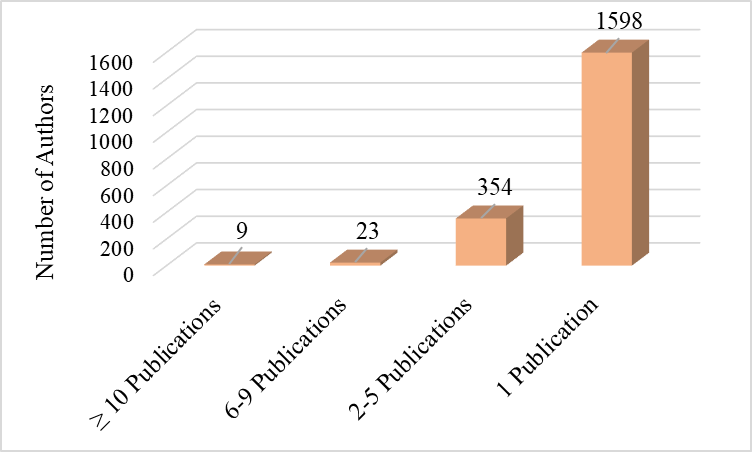


Figure 4 Frequency distribution of authors by publication volume
